# Patient-Derived Xenograft: A More Standard “Avatar” Model in Preclinical Studies of Gastric Cancer

**DOI:** 10.3389/fonc.2022.898563

**Published:** 2022-05-19

**Authors:** Mingtang Zeng, Chao Pi, Ke Li, Lin Sheng, Ying Zuo, Jiyuan Yuan, Yonggen Zou, Xiaomei Zhang, Wenmei Zhao, Robert J. Lee, Yumeng Wei, Ling Zhao

**Affiliations:** ^1^Key Laboratory of Medical Electrophysiology, Ministry of Education, School of Pharmacy of Southwest Medical University, Luzhou, China; ^2^Luzhou Key Laboratory of Traditional Chinese Medicine for Chronic Diseases Jointly Built by Sichuan and Chongqing, The Affiliated Traditional Chinese Medicine Hospital of Southwest Medical University, Luzhou, China; ^3^Central Nervous System Drug Key Laboratory of Sichuan Province, Southwest Medical University, Luzhou, China; ^4^Department of Comprehensive Medicine, The Affiliated Traditional Chinese Medicine Hospital of Southwest Medical University, Luzhou, China; ^5^Clinical Trial Center, The Affiliated Traditional Chinese Medicine Hospital of Southwest Medical University, Luzhou, China; ^6^Department of Spinal Surgery, The Affiliated Traditional Chinese Medicine Hospital of Southwest Medical University, Luzhou, China; ^7^Luzhou Key Laboratory of Traditional Chinese Medicine for Chronic Diseases Jointly Built by Sichuan and Chongqing, Institute of Medicinal Chemistry of Chinese Medicine, Chongqing Academy of Chinese MateriaMedica, Chongqing, China; ^8^Division of Pharmaceutics and Pharmacology, College of Pharmacy, The Ohio State University, Columbus, OH, United States

**Keywords:** gastric cancer, tumor heterogeneity, patient-derived models, interfering factors, applications

## Abstract

Despite advances in diagnosis and treatment, gastric cancer remains the third most common cause of cancer-related death in humans. The establishment of relevant animal models of gastric cancer is critical for further research. Due to the complexity of the tumor microenvironment and the genetic heterogeneity of gastric cancer, the commonly used preclinical animal models fail to adequately represent clinically relevant models of gastric cancer. However, patient-derived models are able to replicate as much of the original inter-tumoral and intra-tumoral heterogeneity of gastric cancer as possible, reflecting the cellular interactions of the tumor microenvironment. In addition to implanting patient tissues or primary cells into immunodeficient mouse hosts for culture, the advent of alternative hosts such as humanized mouse hosts, zebrafish hosts, and *in vitro* culture modalities has also facilitated the advancement of gastric cancer research. This review highlights the current status, characteristics, interfering factors, and applications of patient-derived models that have emerged as more valuable preclinical tools for studying the progression and metastasis of gastric cancer.

**Graphical Abstract:**
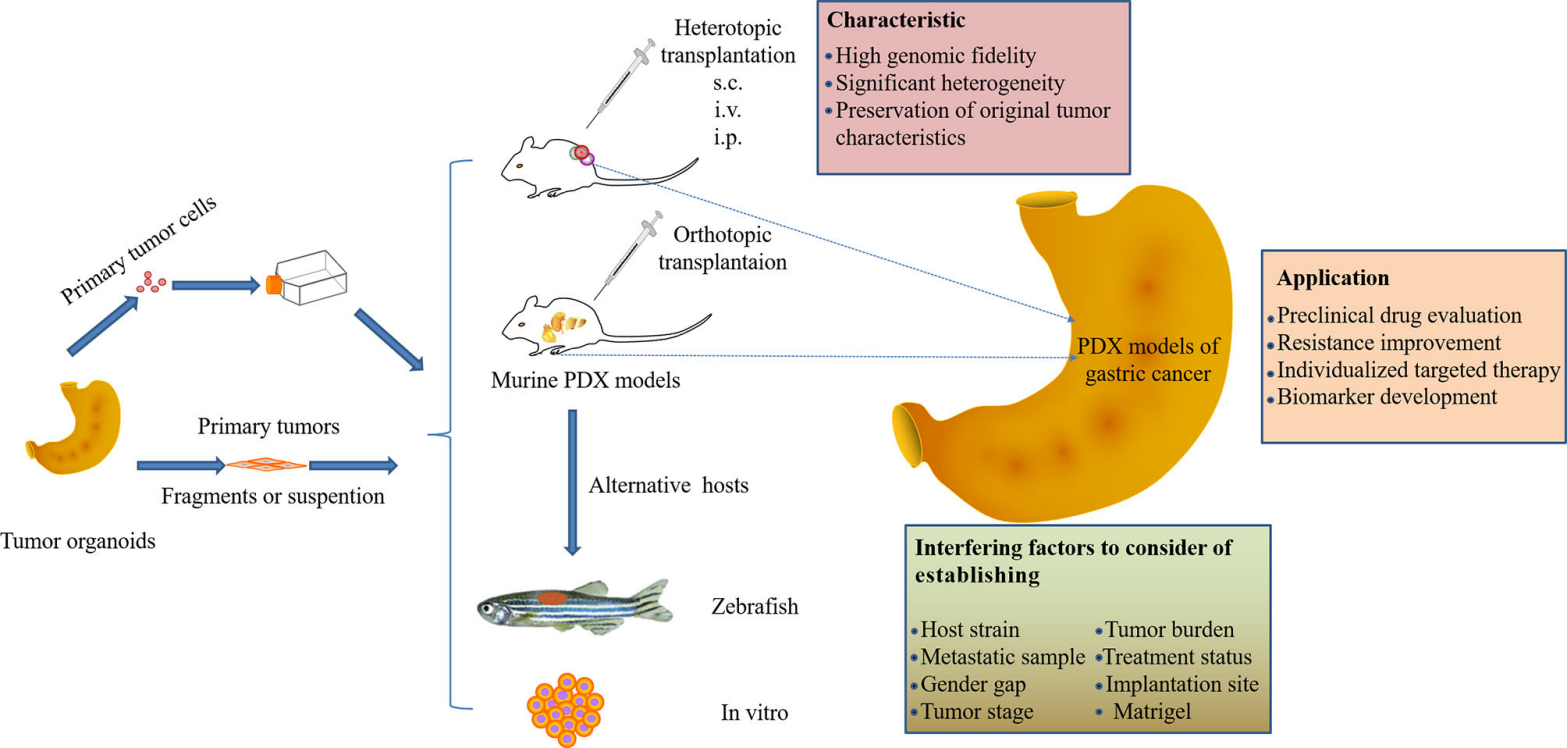


## Introduction

Efforts to treat cancer have improved our understanding of the disease. Cancer, as a heterogeneous disease, has led to various therapeutic outcomes in clinical practice. Gastric cancer (GC) is the fifth most common cancer worldwide and the third most common cause of cancer-related death in humans (1). In fact, as a malignant tumor of the digestive system, gastric cancer is often affected by many factors such as ethnicity, diet, and infectious sources (e.g., Helicobacter pylori), and its incidence and mortality rates vary according to geographical regions (2).

Due to the lack of specific symptoms, defined biomarkers, and diagnostic methods at the early clinical stage, gastric cancer is mostly of middle to late stage when detected. The overall prognosis of patients with gastric cancer remains poor, despite improvements in multidisciplinary and multimodal treatment modalities (surgery, chemotherapy, radiotherapy, etc.). Most gastric cancer patients have a median survival of only 8-10 months, and the 5-year overall survival rate is less than 30% (3, 4). The accumulation of multiple genetic alterations and genetic backgrounds in patients leads to a more diverse tumor phenotype and heterogeneity, which is also associated with the tumor microenvironment (5, 6). Therefore, gastric cancer can be classified into several subtypes based on its molecular characteristics, rather than a single disease. In order to improve treatment efficacy, the subtype and biological heterogeneity of gastric cancer need to be defined earlier by histopathology, thereby affecting the disease response to treatment. Despite advances in therapeutic strategies, most phase III clinical trials have failed due to a lack of efficacy (7, 8). An important factor for failure in clinical trials is the inadequate biology of preclinical models in which drugs are developed or tested, resulting in the inability to predict therapeutic efficacy in humans (9, 10). Fortunately, the advent of a gastric cancer patient-derived xenograft (PDX) model that is established by transplanting fresh tumor tissue or cells from a patient into immunodeficient mice, is critical for improving our understanding of the genetic and molecular etiology of this disease, developing and validating effective therapies as well. In recent years, patient-derived xenograft models have emerged as the most suitable preclinical models of gastric cancer, reproducing the heterogeneous and complex tumor microenvironment of gastric cancer. For example, Chen et al. utilized targeted next-generation sequencing, *in situ* hybridization, and immunohistochemistry to analyze the genomic variations and molecular profiles of 50 gastric cancer PDX models. The majority of PDX models exhibited the same histopathological and molecular features as the primary tumors, and several potential drug targets were validated, such as EGFR, Met, and CCNE1 (11). More understanding about the biological behavior and intrinsic subtypes of gastric cancer has been explored through PDX models, especially the human epidermal growth factor receptor-2 (HER2)-amplified subtype of gastric cancer confirmed HER2 as the first validated therapeutic target for esophageal cancer. These studies highlighted the importance of identifying potential therapeutic targets and developing targeted therapies, and also reflected the significance of patient-derived xenograft models with clinical characteristics for gastric cancer research.

**Figure 1:**
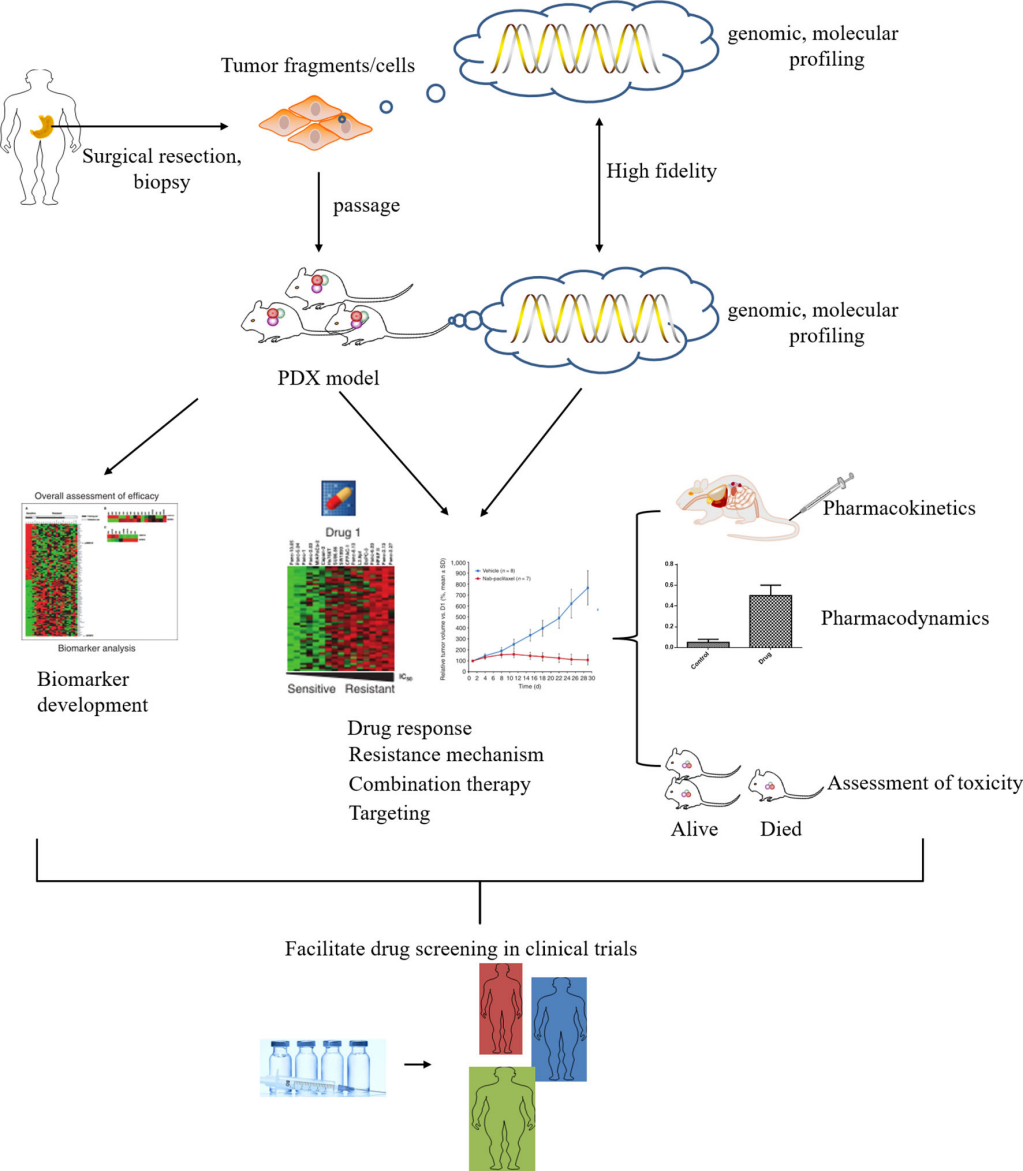
The utility of patient-derived xenograft (PDX) models in gastric cancer research. The gastric cancer tissue or cells of the surgically resected patient was directly implanted into immunodeficient mice, and the fidelity of the model was analyzed. The GCPDX model can be used for biomarker development or novel drug evaluation, etc. Promising candidates may enter clinical trials for evaluation to determine the optimal treatment for patients, enabling personalized medicine.

Although great progress has been made in PDX models of digestive system tumors, the study of PDX models of gastric cancer still needs further exploration. This review summarizes the advantages of patient-derived models as the most appropriate preclinical models of gastric cancer, which maintain high concordance with primary tumors in terms of histopathological features, gene expression, and response to drugs. The application of this model on the identification of biomarkers, the screening of clinical drugs, and precision treatment for gastric cancer will definitely advance the clinical treatment of gastric cancer (**Figure 1**). Special attention is also given to the interfering factors that need to be considered when establishing gastric cancer PDX models to accelerate the translation to the clinic.

## GC Models in Mice

### Patient-Derived Model in Immunodeficient Mice

The development of preclinical models has primarily originated from human cancer cells capable of perpetuating propagation *in vitro*. Studies have shown that common gastric cancer cell lines include: ① MGC-803, BGC-823, and OCUM-1 were obtained from primary foci of gastric cancer; ② SGC-7901 was obtained from a perigastric lymph node metastasis; ③ KATO-II and III were obtained from Cancerous Pleural Fluid; ④ HSC-39, HSC-40A were obtained from cancerous ascites, etc. Masakazu et al. reviewed the origin, morphology, biological characterization, and tumorigenic properties of 20 gastric cancer cell lines (12). Gastric cancer cell lines were transplanted into immunodeficient mice and the corresponding models were established by subcutaneous or orthotopic implantation, intravenous or intraperitoneal injection. However, there are specific defects to this model. First, long-term culture can gradually change the properties of cancer cells under selective pressure *in vitro*, leading to the loss of heterogeneity characteristics of proto cancer cells. Second, cell line models lack a tumor microenvironment. In addition to cancer cells, the microenvironment includes the surrounding lymphatics, capillaries, Stromal cells (immune cells and cancer-associated fibroblasts), other normal cells, extracellular matrix (ECM), and various signaling molecules. Changes in microenvironmental conditions play irreplaceable roles in the growth and maintenance of cancer cells (13). For example, cancer-associated fibroblasts promote the proliferation and invasion of primary gastric cancer, while contributing to tumor development (14–16).

Another alternative to conventional cell line culture is the transplantation of cell lines that are extracted from primary gastric cancer tissues. Fresh specimens of gastric cancer in humans undergo primary cell culture after mechanical isolation to obtain viable tumor cells, which was followed by early passages to establish a gastric cancer cell-line derived xenograft (GCCDX). As a patient-derived xenograft, the GCCDX model is more heterogeneous and fidelity compared to long-term cultured cells, and simulates the characteristics of the primary gastric cancer cell population to a greater extent, because it may contain tumor-associated fibroblasts. However, this cell model generally does not retain other microenvironmental components of primary gastric cancer, and the consistency with the primary tumor may change. From 17 gastric cancer cases, four cell lines were established by directly culturing cells from ascites fluid, and another three cell lines were established by subcutaneously engrafting the primary cultured cells. However, there was some inconsistency between primary and CDX tumors. Among 24 cases in which CDXs were obtained from resected tissue, only about half were of the same differentiation grade. It is noteworthy that more than half of the primary tumors that show differentiated histology turn out to be poorly differentiated adenocarcinoma in CDXs. In addition, this study compared histology between primary tumors and another patient-derived xenograft (PDX) model obtained by implanting tumor biopsies or tissues into immunodeficient mice. All of the 35 PDX models established were all adenocarcinomas, 28 of which had the same histological differentiation grade between primary and PDX tumors. Concordance between primary and PDX tumors was statistically significant (p<0.01). Although the remaining seven cases were classified as different histological differentiation grades, five cases showed mixed differential grades in either primary or PDX tumor lesions. Therefore, most cases (94.3%, 33/35) shared the same histology, at least in some regions, in both primary and PDX tumors (17).

**Figure 2:**
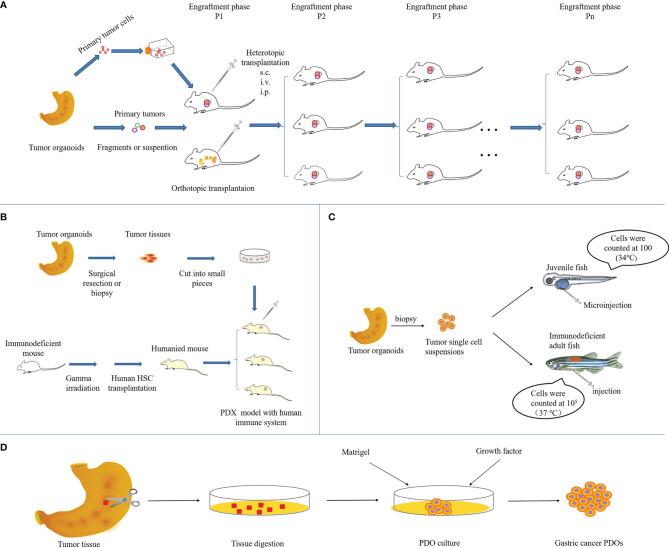
Overview of the methodology to establish GCPDX in **(A)** immunodeficient mice, **(B)** humanized mice, **(C)** zebrafish, and **(D)** *in vitro*.

It follows that tumors formed in PDX models obtained by implanting tumor biopsies or tissues into immunodeficient mice, in addition to having high genomic fidelity, are histologically and genetically closer to the patient’s original tumor, allowing for more accurate prediction of the corresponding treatment regimen (18–20). The direct implantation of tumor specimens avoids culture time and genetic changes for *in vitro* proliferation. At present, most of the established gastric cancer PDX models are subcutaneous transplants, which are quite different from the primary environment of gastric cancer and few metastases occur. However, the benefits are easier to manipulate and easier to monitor tumor growth (21, 22). Patient-derived orthotopic xenografts (PDOX) provide a tumor growth microenvironment that more closely resembles the real situation in patients, which is conducive to tumor occurrence and metastasis (23, 24), while not conveniently monitoring tumor progression (25–27). Sicklick et al. conducted a study on the orthotopic PDX model of gastrointestinal tumors and found that the engraftment success rate of gastric wall tumors can reach 50%, which is favorable for the occurrence of metastasis (28, 29). The establishment method of subcutaneous and orthotopic GCPDX is shown in **Figure 2A**. After the tumor tissue is successfully established in mice, tumor tissues can be continuously transplanted into subsequent mouse offspring, providing a sufficient number of tumors for a variety of applications and statistical analysis. Of course, molecular or genetic phenotypes do not change after many generations. Studies have shown that states and gene phenotypes close to primary patient tumors in a GCPDX were also maintained for 12 generations in mice, with no conversion occurring (30). Moreover, the latency of xenografts in GCPDX is as long as 160 days or even longer (31). The success rate of engraftment of primary tumor specimens into gastric cancer PDX models is approximately 10.7% - 60%, with many influencing factors, which are elucidated in detail later in the review (32).

### Genetically Engineered Mouse Model

Gastric cancer PDX models transplanted in mice run the risk that human-derived stromal components may be progressively assimilated by the mouse due to the altered growth environment, with gradual differences between the transplanted and primary tumor tissues (33, 34). This discrepancy will affect the reliability of research results, so how to avoid or reduce this loss of human-derived stroma is of particular importance. With the development of genetic engineering and molecular biology techniques in recent years, useful information has been obtained in the study of genetically engineered mouse models. Mutations in the p53 gene are one of the most common molecular events in human cancers. A recent study generated mice (DCKO) with double conditional knockout (loss of E-cadherin and loss of p53) by crossing Atp4b-Cre mice with Cdh1fl/f1 and p53fl/fl mice. The DCKO mice showed phenotypes of loss of cell polarity for parietal cells and protein pump negative atypical foci, which ultimate progression to intramucosal cancer and invasive cancer (35). Similarly, loss of E-cadherin and Smad4, as well as loss of p53, induced gastric cancer in mice, recapitulating human diffuse gastric cancer (36). However, another study reported that silencing of E-cadherin in a transgenic mouse model did not result in gastric malignancy, suggesting that loss of E-cadherin may induce precancerous lesions in the gastric mucosa, but may not be sufficient to induce tumorigenesis (37). It follows that the techniques required by the model and the corresponding success rates were not ideal. More notable is that the time of tumor initiation and progression was more than 12 months in transgenic mice by gene knockout or alteration. In addition, monitoring tumor progression requires specialized equipment for imaging that is not available in many laboratories, and the maintenance required of the model after its establishment is costly (25). Most importantly, tumors are derived directly from mice and may not accurately reproduce the heterogeneity of human tumors. Therefore, this model is not the best option to solve the above problems.

### Patient-Derived Model in Humanized Mice

In order to better avoid reducing the loss of this humanized stroma, some scholars have used “humanized mice” to obtain a human immune microenvironment for reconstitution and to establish “humanized PDX models” after transplanting human tumors. For example, humanized immune-reconstituted mice were established by transplanting human embryonic thymus tissue and CD34 + hematopoietic stem cells into NOD/SCID mice (38). This NOG mouse with a humanized immune system is further integrated into the PDX model of gastric cancer, which can mimic the humanized microenvironment and provide human immune cells required for tumor growth (shown in **Figure 2B**) (23). Such models are not only able to provide an immune microenvironment similar to the growth of human tumors, but also address the problem that conventional PDX models cannot be used for antitumor immune drug evaluation due to the use of immunodeficient mice. At present, several strains of immunodeficient mice have been utilized to produce humanized mice: the NOD.Cg-PrkdcscidIl2rgtm1WjlTg (CMV IL-3, CSF2, KITLG)1Eav/MloySzJ (also known as NSG-SGM3) mice, the NOD, B6.SCID Il2rγ−/−KitW41/W41(NBSGW) mice, and the human SIRPA and IL15 knockin(SRG-15) mice (39–41).

Conventional humanized mouse models suffer from incomplete replacement of the hemato-lymphoid system and inefficient human myelopoiesis (42). As humanized mice develop, cytokine humanized mice will become the next generation of humanized mice. A so-called cytokine humanized mice (MISTRG mice) combine genetic preconditioning and cytokine-mediated support by knocking in gene replacement, removing mouse cytokine-encoding genes and replacing them with their human counterparts (40, 43). In such mice, the level of human hematopoietic engraftment in organs such as the bone marrow (BM) is significantly higher. In addition, human phenotypically defined HSPCs in BM, T cells in the thymus, and myeloid cells in nonhematopoietic organs are present at higher levels of engraftment, approaching levels in the human system (44). In this way, human innate and adaptive immune responses to diseases such as COVID-19 and myelodysplastic syndromes have been faithfully recapitulated (45, 46). To pre-evaluate novel HSC-mediated gene therapy approaches, enabling more facile and less costly evaluation of promising strategies, Radtke et al. developed the first “monkeyized” mouse xenografts *via* MISTRG mice model (47). Although there are few studies on cytokine humanized PDX models for gastric cancer, these models will provide an unprecedented platform for gastric cancer immunology and personalized medicine research, with the continuous improvement of the humanized PDX model and precise expression of the human biological system.

## Alternative Hosts in GC Patient-Derived Models

### Patient-Derived Model in Zebrafish

In addition to transplanting tumor tissue from patients into mice, zebrafish is also a powerful and genetically tractable model to study human malignancies (**Figure 2C**). This model demonstrates a high level of physiological and genetic similarity to mammals and closely mimics the clinical environment, favoring natural history surveillance of tumors (48). In recent years, the value of zebrafish PDX (ZPDX) models has become apparent (49–51). Wu et al. xenografted two human gastric cancer cell lines (AGS and SGC-7901) into zebrafish embryos (64% engraftment rate), and the *in vitro* and *in vivo* sensitivity to 5-FU was examined in the model. Xenotransplantation of primary cells from 14 human gastric cancer tissues into zebrafish embryos enabled the *in vivo* observation of tumor angiogenesis, cell invasiveness, and drug response in a time- and cost-saving manner. Importantly, this study is the first ZPDX model for personalized treatment of gastric cancer (52). Furthermore, Zhai et al. established another patient-derived model of gastric cancer derived from juvenile zebrafish to develop a stable and reliable chemotherapy screening protocol and achieve precise chemotherapy for gastric cancer by optimizing the route of administration, drug dosage, and rhythm. Through the new platform, the group investigated the chemosensitivity of 5-fluorouracil, cisplatin, docetaxel, and doxorubicin in gastric cancer patients (53). Zebrafish cancer models, when combined with large-scale genetic screens and drug discovery platforms, have provided valuable insights into the clonal evolution and heterogeneity of tumors, drug resistance to therapy, invasion, metastasis, hematopoiesis, and transplantation of stem cells (54–61).

The advantages of the zebrafish model are mainly: ① transparent embryos have unique features that are beneficial for exploring tumor development, angiogenesis, invasion, and metastasis, etc (62, 63); ② Easy tracking of fluorescently labeled cells; ③ High reproductive capacity and low costs of feeding and maintenance; ④ Easily microinjected or manipulated in bulk, large-scale and high-throughput cell transplantation studies are possible. The above has been difficult to perform in immunocompromised mouse models (64–68). However, most xenograft experiments in zebrafish recipients are performed at 37°C, a temperature at which studies have reported that the proliferation rate of transplanted human cells or the way to form tumor masses is different from that of immunocompromised NSG mice or human patients (69). As zebrafish xenograft technology has slowly matured, it is critical for the field to move to the clinic and conduct clinical studies (70).

### Patient-Derived Organoid Model

The current xenograft models derived from patients with gastric cancer all require host culture, with ethical concerns. This issue was not addressed until the advent of PDO (patient-derived organoid) model, an extension of a patient-derived xenograft model (**Figure 2D**). PDO models are generally derived from pluripotent stem cells (including embryonic stem cells and induced pluripotent stem cells), adult stem cells, and cancer stem cells (16). Current studies have confirmed that human PDO models can be obtained by surgical resection of specimens, tissue biopsy, etc. (71–73). The isolated cells resulting from tissue fragments are suspended on Matrigel and develop into a three-dimensional matrix of cells. Supplements for long-term expansion of normal gastric cancer PDO models include Advanced DMEM/F12, Glutamine, HEPES (N-2-hydroxyethylpiperazine-N-ethane-sulphonic acid) and cytokines (Wnt3A, R-spondin, Noggin, HEGF, HFGF10, and gastrin), etc. (10, 74). This cultural environment is more similar to the real microenvironment *in vivo*, thereby facilitating the growth of PDO models and the maintenance of associated stem cells (4, 5). Generally, the expansion can be stabilized in a short time (1-2 weeks), thus the inherent heterogeneity and biological behavior of the primary tumor tissue can be well preserved. A study led by Hans Clevers, which demonstrated that PDO models derived from human gastric cancer could be bred in the laboratory for the first time, marked the arrival of an era of utilizing gastric cancer PDO models to study gastric cancer (75). Studies in gastric cancer PDO models performed *in vitro* are able to mimic a range of *in vivo* tumor biological behaviors, such as tumor initiation and progression, transduction of molecular signaling pathways in tumors, development of anti-tumor drugs, and targeted therapy in cancer patients (76). In addition, PDO models are continuously proliferated and passaged, which can be used for direct detection or cryopreservation (15, 72).

PDO models derived from a patient’s tumor cells are characterized by the following parts: (1) Long-term culture remains remarkably gene stable, manifested by the ability to maintain the gene expression profile of the initial tumor over a long period of time, thus reflecting the tumor response to various treatment regimens more realistically; (2) The cultural process of PDO models for tumors is not limited to experimental animals, which does not require ethical concerns. Moreover, the process is time-saving and can be cultured in a single large-scale for high-throughput drug screening; (3) PDO models are generated from the culture of many cells (complex and diverse in origin) and are able to reflect tumor heterogeneity; (4) Low-malignant tumors can also be cultured to generate PDO models, filling the constraints of traditional tumor modeling modalities in low-malignant tumors. Furthermore, for drugs whose targets are not tumor cells, a novel solution is to implant PDO models into mice for drug susceptibility testing (77). This protocol may further refine the design of studies *in vitro* and *in vivo*, and better serve studies on gastric cancer pathogenesis and drug treatment.

## Interfering Factors to Consider in Establishing a Murine GCPDX Model

Studies have shown that the value and feasibility of engrafting PDX models into immunodeficient mice is optimal. To construct murine GCPDX more effectively, the key factors need to be summarized as follows to improve the transplantation rate (**Table 1**).

**Table 1:** Summary of the success rates of GCPDX under different conditions.

Mice Strain	Implantation site	Tumor tissues	Engraftmentrate	Availablemodel	References
NSG	subcutaneous	1mm^3^/fragment	80%	–	(78)
NOD-SCID (F1) BALB/c-nude (Fn)	subcutaneous	3*3*3mm^3^/fragment	–	5	(79)
NOD/SCID	subcutaneous	2*2*2mm^3^/fragment (F1) 3*3*3mm^3^/fragment (Fn)	34.1%	63/185	(31)
BALB/c-nude	subcutaneous	3*10^5^/mL/cell	28.1%	9/32	(80)
NMRI nude	–	1-2 mm^3^/fragment	27%	27/100	(81)
Nude	subcutaneous	2mm^3^/fragment	16.9%	14/83	(30)
SCID			26.9%	32/119	
Nude	subcutaneous/renal capsule	3*3*3mm^3^/fragment	8.0%	6/75	(82)
NOG			10.5%	9/86	
NOG	subcutaneous	1mm^3^/fragment	31.0%	55/177	(17)
NSG			30.0%	3/10	
SCID			22.2%	10/45	

### Host Strain

Nude mice, NOD, SCID, NSG, and other mice are often selected to prepare GCPDX. Yoon et al. reported the successful establishment of 15 GCPDX using nude and NOG mice, and the corresponding success rates of 8.0% and 10.5%, respectively, without significant differences (82). In another study of 207 human GCPDX models, 49 immunodeficient mouse models grew tumors within 3 months after implantation with success rates of 16.9% (nude mice) and 26.9% (SCID mice), respectively. The results confirmed that although there were all trends towards higher success rates using SCID mice, these differences were not statistically significant (30). In addition, Kuwata et al. also evaluated the engraftment rate of NOG, NSG, and SCID mice in GCPDX and found similar success rates for tumor development in the three models: NOG 31.0% (55/177), NSG 30.0% (3/10) and SCID 22.2% (10/45) (17). In fact, a large proportion of scholars would choose BALB/c nude mice for the establishment of the first-generation PDX model to reduce the occurrence of lymphoma, even if other immunodeficient mice are used in subsequent passages (83). However, the degree of immunodeficiency affects engraftment rates, and strains of mice with the most severe immunodeficiencies typically have the highest engraftment rates, such as NMRI nude mice, Bl6/Rag2/GammaC double knockout, or CD-1 mice. Meanwhile, these models are also affected by immunoproliferative diseases, with higher construction costs. It follows that the preparation of GCPDX should be comprehensively considered.

### Type of Sample Being Grafted

Whether the GCPDX can be established is closely related to the number of cancer cells and the size and density of tumor fragments, which can reflect the trends of cancer cells and microcarcinoma tissues at the molecular level (84). For tumor cells of gastric cancer, a higher percentage of tumor cells in a representative tissue was associated with a higher success rate (P = 0.025), and the number of cells was generally adjusted in the interval range of 10^5^-10^7^ according to the actual conditions. Meanwhile, the success rate (38.9%) was higher than that of a single fragment (18.2%) when multiple fragments were implanted in a single animal (82). Tumors were generally inoculated into mice to grow the next generation of PDX when the volume of the primary tumor approached 1000 mm3 (17, 78). Excessively large tumors easily affect the survival state of mice, resulting in the lack of nutrition of tumor cells, and the transplantation ability of the tumor is weakened. If the tumor is too small in size, there will be insufficient stromal cells to form the next generation of tumors. The multiple tumor fragments being transplanted are mostly 1-2 mm in diameter (85–87). Excessive tumor volume easily affects the accuracy of tumor transplantation, leading to a shift in the transplantation position, reducing the transplantation rate. On the contrary, tumor fragments that are too small may not adequately reflect the heterogeneity of the primary tumor, thereby affecting the predictive value of PDX in drug screening.

### Treatment Status

Whether patients receive treatment before tumor resection will hinder the successful establishment of GCPDX remains controversial. In a recent study, 63 PDX models were successfully established using 185 fresh gastroscopic biopsies of gastric cancer and passaged to maintain tumors *in vivo*. The engraftment rate of biopsies inoculated with prior chemotherapy (52.1%, 37/71) was higher than that of biopsies obtained after chemotherapy (21.9%, 25/114) (31). Moreover, Kuwata et al. demonstrated that the establishment success rate of PDXs from patients who received chemotherapy was higher than that from patients who did not receive chemotherapy in 35 GCPDX [26.4% (9/34) vs. 13.1% (26/198)], although the difference was not statistically significant (17). The reason for the existence of this phenomenon may lie in the high degree of tumor differentiation in some patients with gastric cancer who receive chemotherapy, and the malignant tumors are invasive and metastatic even after chemotherapy, thus the staging and differentiation degree of gastric cancer need to be considered. For example, 232 gastric cancer tissues were subcutaneously transplanted into immunodeficient mice and 35 GCPDX were successfully established. The differentiated adenocarcinomas (DAS, 19.4%) were more effective than poorly differentiated adenocarcinomas (PDAs, 10.8%). The investigation revealed that the metastatic status of pathological lymph nodes was significantly correlated with the success rate (17).

### Metastatic Sample

GCPDX can be successfully created from clinical biopsy specimens of patients with metastatic or unresectable gastric cancer (88), and metastatic cancers have a higher engraftment rate than PDX models. In one study, biopsy specimens from 29 patients were used for the engraftment of PDXs. The results demonstrated the highest engraftment rate for gastric and gallbladder cancer specimens (100%), compared to 33% and 29% for PDAC and cholangiocarcinoma, respectively. PDX models created from metastatic biopsies present higher engraftment rates compared to tumor tissue from unresectable primaries (69% vs. 15.4%, P = 0.001), and PDX models have a higher engraftment rate when biopsied during surgical procedures compared to image guidance (73% vs. 14%, P = 0.003) (88). In addition, shorter ex vivo times of isolated tissue or biopsied samples facilitate engraftment of tumors. In a recent study, the ex vivo times of successful cases of GCPDX differed greatly from unsuccessful cases (median time for successful cases was 75 minutes vs. 135 minutes for unsuccessful cases, P = 0.003). Similarly, shorter overall procedure time was associated with engraftment success (123 min for successful engraftment vs. 167 min for unsuccessful engraftment, P = 0.01) (82).

### Subtypes

The 2014 Cancer Genome Atlas (TCGA) group classified gastric cancer into four subtypes: chromosomal instability (CIN), microsatellite instability (MSI), genomic stability (GS) and EBV-positive (Epstein-Barr Virus positive, EBV+) types (89). In one study, Peille et al. showed that not all molecular subtypes of gastric cancer were established in 27 PDX models, with PDXs predominantly developed from MSI, CIN subtypes. In contrast, PDXs were rarely or not developed from the subtypes of EBV and GS. Gastric cancers of the MSI subtype accumulate a large number of mutations that may confer some adaptability to tumor cells to grow in a new microenvironment (the immune-compromised mice). Other subtypes, such as GS, may require additional growth factors to promote proliferation (81). Again, histologic type and MSI-H status were associated with high engraftment, as well as high RTK/RAS (a common molecular alterations in subtype of CIN) copy-number variation (90). MSI, and CIN subtypes were the most common in the clinical diagnosis of gastric cancer, accounting for approximately three-quarters (91). EBV-positive gastric cancer accounts for about 10%, but its establishment in PDX models is uncommon, although more than 30% of PDX generated from gastric cancer samples will develop EBV-positive lymphoma (92). However, Soldan et al. investigated the efficacy of the EBNA1 inhibitor VK-1727 in xenografts through EBV-positive (SNU719 and YCCEL1) GC cell lines (93). In addition, a recent study demonstrated that the stomach-specific and inducible Cre recombinase mouse line Anxa10-CreER^T2^ allows the modeling of different known subtypes of gastric cancer, although in this study only the evaluation of CIN and GS gastric cancer subtypes was achieved with emphasis (94).

### Gender Gap

Unlike hormone-dependent prostate cancer and breast cancer, most other types of tumors do not require the addition of sex hormones such as estradiol or testosterone to improve transplantation efficiency. However, mouse models of gastric cancer tissue or intestinal gastric cancer from male patients are more likely to be successfully established (95). In part, an androgen receptor (AR) was demonstrated to directly regulate miR-125b expression. The AR- miR-125b signaling pathway inhibits apoptosis and promotes proliferation, and therefore can promote transplantation efficiency.

Finally, factors such as implantation site, tumor stage, or whether Matrigel was used are also potential conditions for the successful establishment of GCPDX, which need to be further studied (82).

## Murine GCPDX Models in Cancer Research

### Preclinical Drug Evaluation and Resistance Improvement

One of the major problems in oncology drug development is the low success rate of new drugs, with only 5% of preclinical anticancer drugs eventually approved for clinical treatment. Many anticancer drugs failed due to lack of efficacy in phase II and III clinical trials and wasted a lot of resources, mainly because of the low predictive value of conventional preclinical models for screening new formulations for clinical development (96). As a preclinical model with high predictive value, the PDX model plays an irreplaceable role in the preclinical screening of new anticancer drugs. For example, Huynh et al. evaluated the antitumor activity of oral regorafenib in eight GCPDX. Regorafenib reduced tumor angiogenesis by 3-fold to 11-fold compared with the control group in all models, decreased tumor proliferation by 2-fold to 5-fold in six models, and induced apoptosis in seven models. The results demonstrated that regorafenib was effective in GCPDX of different histological subtypes. The inhibition of tumor growth, angiogenesis, and proliferation of tumor cells by the drug was observed in almost all models, providing support for further clinical studies in gastric cancer (97). In another study, a novel vascular endothelial growth factor (VEGF) blocker with anti-angiogenic properties, FP3 (KH902/KH903), caused an effect on tumor blood vessels in patient-derived models of gastric cancer. The intervention of FP3 decreased the vessel density and inhibited vessel sprouting in the model. Since the degree of degeneration of pericytes of vessels was different from that of endothelial cells, pericytes of vessels surviving on tumor vessels after FP3 treatment exhibited a more normal phenotype. Therefore, FP3 has a direct, rapid anti-angiogenic effect on tumor vasculature, which is mainly achieved by regression of tumor vasculature, inhibition of new and recurrent vessel growth, and normalization of existing tumor vasculature (98). The antitumor effect of luteolin was assessed in two GCPDX models which overexpressed cMet. The results demonstrated that luteolin exerted significant antitumor effects in the cMet-overexpressing xenograft model of gastric cancer through cMet/Akt/ERK signaling, so it may serve as a potential therapeutic option for cMet-overexpressing gastric cancer (99). TR1801-ADC was reported to have excellent efficacy and significant antitumor activity in 90% of xenograft models derived from patients with gastric, colorectal, and head and neck cancers: 7 of 10 gastric models, 4 of 10 colorectal cancer models, and 3 of 10 head and neck cancer models showed complete tumor regression after a single administration (100).

In addition to single drug administration, the potential utility of GCPDX is reflected in combination therapy to address the issue of resistance to clinical anti-gastric cancer drugs. Studies have shown that trastuzumab in combination with paclitaxel can still exert more potent antitumor efficacy than each drug alone in the GCPDX of trastuzumab-resistant, providing evidence that paclitaxel is still beneficial in treating trastuzumab-resistant tumors (101). Another report validated that the co-activation of MET and FGFR2 in the GCPDX increased the drug resistance to targeted therapy, possibly due to the activation of multiple growth and survival signaling pathways. Therefore, the combination therapy of MET and FGFR2 inhibitors may be a promising strategy to treat the inherent resistance of MET inhibitors in gastric cancer cases containing MET and FGFR2 amplification (102). Other discussions using GCPDX to demonstrate drug efficacy are summarized in **Table 2**, and the potential targets of different drugs in the model are distinguished.

**Table 2:** GCPDX models as preclinical research tools to evaluate drug efficacy.

Study Type	Mice Strain	Tumor Location	Drug Target (s)	Drug (s) Evaluated	Observation	References
Efficacy	NOD/SCID	subcutaneous	CDK12, PAK2	Procaterol	CDK12 and PAK2 as novel therapeutic targets for human gastric cancer	(103)
Efficacy	Nude	subcutaneous	RSK2	Carnosol	Carnosol is an RSK2 inhibitor for treating gastric cancer	(104)
Precision medicine	Nude	subcutaneous	c-Met	Volitinib	Volitinib as a therapeutic option for patients with GC tumors harboring amplified c-Met	(105)
Precision medicine	NSG	subcutaneous	ERBB2 and MET	Afatinib + MET inhibitor	Sensitivity and resistance of trastuzumab-resistant GC cancer to therapy were associated with EGFR/ERBB2 amplification and MET amplification	(106)
Precision medicine	F1: NOD-SCID Fn: nude	subcutaneous	HER2^+^	HER2 antibody + Herceptin	Matching rate of above 80% between original patient tissues and p5 PDX tissues	(79)
Efficacy	BALB/c nude	subcutaneous	VEGF, MMP-7, EGFT, Ki-67 and PCNA	Trastuzumab + Cetuximab	A cancer therapy specific to a stage III GC patient	(107)
Efficacy	Athymic nude	subcutaneous (Matrigel)	Wnt/β-catenin target genes (AXIN2, MYC, and LGR5)	2,4-diamino-quinazoline	Wnt-signaling pathway is a druggable therapeutic target in the treatment of GC	(108)
Efficacy	BALB/c nude	subcutaneous	JAK2/STAT3	CYT997	Inhibiting JAK2/STAT3 pathway is a critical modulator of CYT997-induced autophagy and apoptosis in gastric cancer	(109)
Precision medicine	BALB/c nude	subcutaneous (Matrigel)	HER2 heterogeneity that is unresponsive to T-DM1	DS-8201a	Favoring treatment of HER2 heterogeneous tumors unresponsive to T-DM1	(110)

### Individualized Targeted Therapy

Gastric cancer is highly heterogeneous with poor sensitivity to conventional chemotherapeutic agents and poor prognosis, for which individualized targeted therapy is of particular importance. At present, the targets of gastric cancer drugs are mainly concentrated at the molecular level, which can be specific antigens or receptors on the surface of tumor cells or specific antigens or receptors on the surface of tumor neovascular endothelial cells. For example, ① trastuzumab is the first drug to be applied in targeted therapy of gastric cancer, which targets HER2 (111); ② ramucirumab was approved by the U.S. FDA in 2014 for the treatment of advanced gastric cancer, which is an anti-VEGF-R2 monoclonal antibody that inhibits the binding of this receptor to VEGF to inhibit its activation and enable the angiogenesis effect of VEGF could not be achieved, resulting in multiple effects of anti-tumor blood vessels (112). Due to the high specificity of targeted therapy, the same targeted drugs may have large differences in efficacy for patients with different genotypes. The screening of therapeutic targets is the core of targeted therapies, which are often the result of high expression of novel or pre-existing genes that arise after tumorigenesis is mutated. Here, people analyzed and explored potential therapeutic targets based on the characteristic PDX models of advanced gastric cancer (AGC). The genomic variations and molecular profiles of 50 PDX models from AGC patients were analyzed by targeted next-generation sequencing, *in situ* hybridization and immunohistochemistry. Each PDX model has separate histopathological and molecular features, and alterations in MAPK, ErbB, VEGF, mTOR, and cell cycle signaling pathways are largely responsible for the PDX model heterogeneity. The present study also validated several potential drug targets and the antitumor activity of targeted agents. Volatinib exhibits strong anti-tumor activity in GCPDX models, with overexpression of MET and phosphorylated MET (p-MET). The EGFR monoclonal antibody BK011 and cetuximab inhibited tumor growth in the GCPDX model with EGFR amplification. Afatinib inhibited tumor growth in GCPDX models through EGFR amplification, EGFR overexpression, or HER2 amplification. Apatinib was more sensitive in GCPDX models with high microvessel density. The CDK1/2/9 inhibitor AZD5438 had superior antitumor activity in two models with the higher copy number of CCNE1 (11). Similarly, another study implanted GC tissues from 32 patients into immunodeficient mice and assessed protein levels or gene amplification of HER2, cMet, and FGFR2 in these tissues using immunohistochemistry and fluorescence *in situ* hybridization. Different anti-tumor efficacy was tested in the PDX model by using targeted inhibitors. Crizotinib and AZD4547 exerted a significant anti-tumor effect only in PDX models with cMet (G30, G31) and FGFR2 (G03) amplification. Interestingly, a synergistic anti-tumor activity of the two drugs was observed in G03 [FGFR2-amplified and cMet non-amplified but IHC (2+)] (80). In conclusion, GCPDX from different patients better preserves the heterogeneity of primary tumor cells, whose properties may vary slightly, partly reflected in the expression of genes. Targeted drugs that are effective on known models, do not necessarily have the same efficacy on models of other patients. When discrepancies arise, sources should be analyzed and studies of genotypes should be carried out to make personalized treatment realistic. In addition, GCPDX with defined molecular characteristics are useful for preclinical studies of targeted drugs, and the results should be verified in larger studies of PDX models or clinical trials.

### Biomarker Development

The clinical diagnosis of gastric cancer remains challenging, and its early-stage patients often present with non-specific symptoms and there is no screening method. However, tumor biomarkers have the potential to aid diagnosis, improve prognosis, and monitor response to treatment. PDX models have contributed greatly to the diagnosis of potential biomarkers related to gastric cancer. In one study, Kasai evaluated the antitumor efficacy of a novel anti-ASCT2 humanized monoclonal antibody, KM8094, which acts as a therapeutic antibody for gastric cancer with neutralizing activity against glutamine uptake. Interestingly, the results observed a correlation between antitumor efficacy and low antigen expression as well as low glutamine uptake levels, suggesting that ASCT2 expression levels may be a potential predictive biomarker for KM8094. They further explored predictive biomarker candidates using multi-omics analysis of a GCPDX, which selected some potential candidates through gene expression and DNA methylation array analysis, including TFF2, MUC13, and ANG (113). In addition, SL1, a LIM homeodomain transcription factor, serves as a biomarker for metastasis in multiple tumors. However, the function and underlying mechanism of ISL1 in GC have not been fully elucidated. It has been revealed in a GCPDX that a complex between ISL1 and SETD7 (a histone H3K4-specific methyltransferase) can directly bind to ZEB1(Key regulator of epithelial-mesenchymal transition (EMT)) promoter to promote the molecular mechanism of GC metastasis, which demonstrated that ISL1 may be a potential prognostic biomarker for GC (114). Cancer stem cells (CSCs) are a subgroup of cancer cells with self-renewal properties that are responsible for tumor malignancy, progression, and drug resistance. However, studies on specific markers of CSC in GC are still limited. Zhang et al. explored the expression of voltage-dependent calcium channel α2δ1 subunits and the potential of using α2δ1 as a marker for CSCs of gastric cancer. The expression of α2δ1 was analyzed in GC cell lines, PDX and clinical samples of malignant ascites from gastric cancer patients, and the results revealed that the expression level of α2δ1 was significantly different among the three models, while α2δ1+ gastric cancer cells exhibited significant self-renewal properties, including tumorigenic capacity, sphere-forming ability, and asymmetric differentiation potential. Therefore, the author proposes that α2δ1+ gastric cancer cells have CSC properties, and α2δ1 may be an appropriate marker for identifying GCSC (115). Finally, GC is commonly treated with combination chemotherapy using 5-fluorouracil (5-FU) derivatives and platinum, but predictive biomarkers are still lacking. Na group developed PDX models from 31 GC patients and treated them with a combination of 5-FU and oxaliplatin to identify biomarkers associated with responsiveness. The final data suggest that defects in p53 signaling are one of the predictive features of chemoresistance to 5-FU and oxaliplatin. During the development of resistance to chemotherapy with 5-FU and oxaliplatin, cancer cells and the TME work synergistically by activating similar pathways (116). From this, it appears that PDX models have powerful utility in identifying biomarkers of disease progression or treatment response. Contributing to this phenomenon is that PDX models retain the genomic and molecular characteristics of their tumors of origin, thus enabling prospective studies of biomarkers and comparison of preclinical data with clinical outcomes.

## Limitations and Outlook

Gastric cancer PDX models still have limitations. First, the number requirement of models for partial experiments is large-scale, corresponding to the high cost of establishment and maintenance, and the long establishment time hinders the treatment of gastric cancer patients with expected shorter survival. The number of tissues used for implantation is limited, and the vast majority of GCPDX occurs at less than 50%. In order to improve the transplantation rate, a new method was developed that allowed the complete insertion of soft tissue fragments or evenly shredded tissue into the submucosa of the stomach of immunodeficient NOD.Cg-Prkdc Il2rg/SzJ (NSG) mice. Tumors of various tissue types can be used to establish orthotopic gastric cancer models through this completely enclosed transplantation method, without exposing adjacent organs or presenting the risk of cell leakage. This surgical procedure is highly reproducible in generating 48 mouse models with a success rate of 96% and a tumor formation rate of 93% (117). Second, the incidence of developing EBV-related B-cell lymphoma was as high as 68% when PDX models of gastric cancer were generated using severe combined immunodeficient mice NOD/SCID, NSG, or NOD, especially in the F1 generation (33.3%) (82). However, lymphomagenesis can be reduced using nude mice, which do not form lymphoma even when NOD (F2) mice are used in subsequent transplants. The reason is that EVB-infected B cells are in a latent state in the human body, and when tissues are transplanted into severely immunodeficient mice, these cells are prone to be activated to form lymphomas due to the lack of functional immune cells, which is the same mechanism as the formation of lymphomas in clinically immunocompromised patients (118). The presence of functional NK cells in nude mice inhibited the activation of latently infected EVB. Since H.pylori-associated gastritis is highly correlated with gastric carcinogenesis, and the degree of basal inflammation occurs to a significantly higher extent in gastric cancer cases than in other tumors, the incidence of lymphoma in GCPDX is significantly higher than in other tumors (32). The establishment of primary PDX models using nude mice not only ensures the success rate but also avoids the formation of lymphoma. Moreover, the development of this type of lymphoma can be prevented by short-term treatment of rituximab on murine implants without negatively affecting the engraftment of gastric cancer (92). Third, PDX tumors propagate in immunocompromised mice, and most stromal tissues and residual immunocompetent cells (if any) are derived from mice, which could not be considered to have a fully authentic tumor microenvironment. Therefore, the use of PDX models to evaluate drugs that target tumor-interstitial interactions or immune-mediated anti-tumor efficacy is hindered, which can be solved by the above-mentioned “humanized PDX model”. Although humanized mice still have deficiencies, especially in the degree of immune system reconstitution, which may lead to different immunotherapy efficacy. Several types of human hematopoietic cells could not fully differentiate with hematopoietic stem cells in any humanized mouse strain, such as erythrocytes, platelets, neutrophils, NKT cells, and ILC2 (119). To enhance human erythropoiesis and RBC survival in the models, a study reported a novel immunodeficient mouse model with deletion of the fumarylacetoacetate hydrolase gene in MISTRG mice and intrasplenic injection of human hepatocytes (huHep) (120). Finally, metastasis is an important reason why advanced gastric cancer is difficult to cure, and it is of great significance to prepare the corresponding metastasis model. As mentioned in the second part of the article, most of the GCPDX are subcutaneous transplantation, which could not fully simulate the primary environment of gastric cancer and rarely metastasize. Orthotopically transplantation of PDX models can effectively solve this problem, but the technical means required are elevated at the same time. In addition to the establishment of PDX models using primary gastric cancer tissues, there are also reports using metastatic tumor modeling. A novel patient-derived orthotopic xenograft (PDOX) model of gastric cancer liver metastasis evaluated the efficacy of novel combination chemotherapy including gemcitabine (GEM) combined with 5-fluorouracil (5-FU) or oxaliplatin (L-OHP) combined with 5-FU in a standard regimen for liver metastasis. A single tumor fragment was implanted into the liver of nude mice, and patient-derived gastric cancer was successfully established by tumor cell metastasis (121).

## Conclusion

The intra-tumoral and inter-tumoral phenotypes of GC are remarkably heterogeneous and distinct molecular signatures correspond to discriminating therapeutic responses, which are not well reflected in most preclinical models. However, as a promising and innovative preclinical tool, PDXs are more suitable for studying gastric cancer onset, progression, and metastasis (generally orthotopic transplantation), investigating mechanisms of resistance to therapy, and understanding cell proliferation and evolution during tumor growth. Despite the increasing relevance of patient-derived models in GC research and therapy, there are several relevant limitations in patient-derived models due to the lack of human immune cells and stromal cells that contribute to tumor progression by interacting with tumor cells. A humanized PDX mouse model was established to recapitulate human tumor microenvironment-immune cell interactions in PDXs. For a more intuitive view of how gastric cancer develops in a model, zebrafish were adapted into a PDX model, which also offers the advantages of low cost and convenience for large-scale and high-throughput studies. With continued optimization and realization, patient-derived models represent the most promising preclinical models of GC to dissect the multifactorial etiology and progression of tumors. Meanwhile, the interfering factors involved in the establishment efficiency of this model need to be considered, including host strain, transplantation site, distinctions between tissues and cells, staging grade of gastric cancer, and so on, to better drive personalized treatment of GCPDX.

## Author Contributions

All authors contributed to the article and approved the submitted version.

## Funding

This study was supported by the Cooperative Scientific Research Project of Chunhui Plan of the Ministry of Education of China (No. 2020-703), the Youth Science and Technology Innovation Research Team (No. 2021JDTD0008), and the Basic Research Fund (No. 2020YJ0336, 2020YJ0373) of the Science and Technology Department of Sichuan province of China, Science and Technology Innovation Team from Jiucheng Science and Technology Talent Cultivation Plan in Luzhou City (No. 2019-1), and Key Research and Development Projectors of Office of Science & Technology and Talent Work of Luzhou (No. 2021-SYF-26).

## Conflict of Interest

The authors declare that the research was conducted in the absence of any commercial or financial relationships that could be construed as a potential conflict of interest.

## Publisher’s Note

All claims expressed in this article are solely those of the authors and do not necessarily represent those of their affiliated organizations, or those of the publisher, the editors and the reviewers. Any product that may be evaluated in this article, or claim that may be made by its manufacturer, is not guaranteed or endorsed by the publisher.
